# Tuberculosis-Associated Immune Reconstitution Inflammatory Syndrome—An Extempore Game of Misfiring with Defense Arsenals

**DOI:** 10.3390/pathogens12020210

**Published:** 2023-01-29

**Authors:** Ramachandran Vignesh, Pachamuthu Balakrishnan, Hong Yien Tan, Yean Kong Yong, Vijayakumar Velu, Marie Larsson, Esaki M. Shankar

**Affiliations:** 1 Preclinical Department, Faculty of Medicine, Royal College of Medicine Perak, Universiti Kuala Lumpur, Ipoh 30450, Perak, Malaysia; 2 Department of Microbiology, Centre for Infectious Diseases, Saveetha Dental College and Hospitals, Saveetha Institute of Medical and Technical Sciences (SIMATS), Chennai 600077, Tamil Nadu, India; 3 School of Traditional Chinese Medicine, Xiamen University Malaysia, Sepang 43900, Selangor, Malaysia; 4 Laboratory Centre, Xiamen University Malaysia, Sepang 43900, Selangor, Malaysia; 5 Division of Microbiology and Immunology, Yerkes National Primate Research Center, Emory University, Atlanta, GA 30322, USA; 6 Department of Biomedicine and Clinical Sciences, Linköping University, 58185 Linköping, Östergotland, Sweden; 7 Infection and Inflammation, Department of Biotechnology, Central University of Tamil Nadu, Thiruvarur 610005, Tamil Nadu, India

**Keywords:** ART, biomarkers, HIV, inflammation, IRIS, tuberculosis

## Abstract

The lethal combination involving TB and HIV, known as “syndemic” diseases, synergistically act upon one another to magnify the disease burden. Individuals on anti-retroviral therapy (ART) are at risk of developing TB-associated immune reconstitution inflammatory syndrome (TB-IRIS). The underlying inflammatory complication includes the rapid restoration of immune responses following ART, eventually leading to exaggerated inflammatory responses to MTB antigens. TB-IRIS continues to be a cause of morbidity and mortality among HIV/TB coinfected patients initiating ART, and although a significant quantum of knowledge has been acquired on the pathogenesis of IRIS, the underlying pathomechanisms and identification of a sensitive and specific diagnostic marker still remain a grey area of investigation. Here, we reviewed the latest research developments into IRIS immunopathogenesis, and outlined the modalities to prevent and manage strategies for better clinical and diagnostic outcomes for IRIS.

## 1. Introduction

Tuberculosis (TB) continues to be the most prevalent cause of morbidity and mortality among people living with HIV/AIDS (PLWH) [1,2,3]. The lethal combination of TB and HIV is known as a “syndemic”, referring to diseases acting synergistically and thereby amplifying the disease burden [4,5]. About one-quarter of the world’s population is estimated to have latent TB infection [6]. While only 10% of HIV-negative people have the chance of progressing to active TB, PLWH are 18 times more likely to develop the active disease [6,7]. Coinfection with HIV can also increase the transmission of TB by disruption of the granulomatous lesions containing the *Mycobacterium tuberculosis* (MTB) bacilli [8]. Furthermore, through various cellular mechanisms, TB infection, in most cases, impacts the disease progression of HIV by augmenting the replication and expansion of reservoir sites [5,9].

Wider access to anti-retroviral therapy (ART) has been pivotal in the fight against HIV and TB syndemics in regions badly hit by these infections. The ART roll-out has been reported to lower the risk of acquiring TB by about 58–80% and lower the TB mortality among PLWH [10]. However, on the negative side, PLWH starting on ART are at risk of developing TB-associated immune reconstitution inflammatory syndrome (TB-IRIS) [11,12,13,14,15]. The immunological mechanism underlying this acute inflammatory complication includes the rapid restoration of immune responses following ART, eventually leading to exaggerated inflammatory responses to MTB antigens [11].

Studies have demonstrated higher incidence of TB-IRIS among PLWH with a low CD4^+^ T cell count at the baseline [11,16,17,18,19,20]. Before the World Health Organization (WHO) released the “Test and Treat” policy in 2005, the median CD4+ T cell counts at the time of ART initiation in African and Asian countries had always been lower [21,22,23,24,25,26]. Although all PLWH in India are eligible to start ART based on the “Test and Treat” policy introduced in 2017, only about 49% of Indian PLWH are estimated to be on ART [26]. Cohort studies from low and middle income countries) including India and African countries have reported incidence rates of TB-IRIS as high as 19 to 57% [11,17,27,28]. 

## 2. TB-IRIS: Types and Definitions

**Figure 1:**
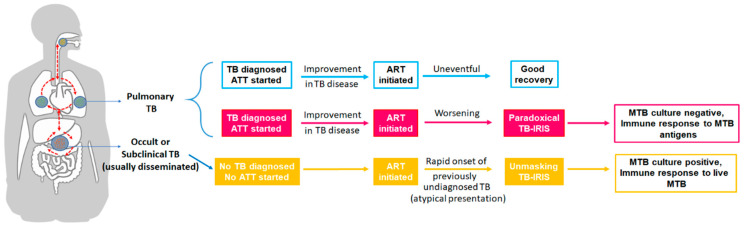
Schematic representation of HIV/TB-immune reconstitution inflammatory disease. Following the initiation of anti-retroviral treatment (ART), some individuals with HIV and active TB disease display strong immune activation resulting in new or recurrent TB symptoms referred to as paradoxical TB-IRIS [11,32]. Paradoxical TB-IRIS usually develops during the first four weeks of ART and results in the flaring up of TB symptoms such as a new infiltrate, serous effusions, worsening of existing lesions, and soft tissue abscesses [33]. Unmasking TB-IRIS represents a subcategory of ART-associated TB observed in patients with undiagnosed or subclinical TB before initiation of ART. In unmasking TB-IRIS, a subclinical TB infection remains undiagnosed until ART-induced immune reconstitution elicits an exaggerated presentation of the disease [35,36]. Unmasking TB-IRIS usually occurs within three months of starting ART with high levels of clinical manifestations including lymphadenitis, abscess, and respiratory failure [37].

Numerous opportunistic infections (OIs) including mycobacterial, fungal, viral and parasitic infections are associated with IRIS. Fungal infections such as cryptococcosis, pneumocystis pneumonia, histoplasmosis, and viral infections including herpes simplex virus, herpes zoster virus, hepatitis B and C virus as well as parasitic infections such as strongyloidiasis and toxoplasmosis are commonly associated with IRIS [29]. Of those infections associated with IRIS, tuberculosis, being the most common OI, remains the most common form of IRIS [30,31]. Shortly after the initiation of ART, some PLWH with active TB present with strong immune activation resulting in new or recurrent TB symptoms referred to as paradoxical TB-IRIS [11,32]. Paradoxical TB-IRIS usually develops during the first four weeks of ART and generally gives rise to an elevation of TB symptoms such as a new infiltrate, serous effusions, worsening of existing lesions, and soft tissue abscesses [33]. This complicates the therapeutic management of HIV-associated TB in about 18% of cases, thereby causing substantial morbidity with about 25% requiring hospitalization [11,34]. The other form of IRIS, i.e., the unmasking TB-IRIS, is a subcategory of ART-associated TB observed in patients with undiagnosed or subclinical TB before the initiation of ART. Unmasking TB-IRIS refers to the scenario wherein a subclinical TB infection remains undiagnosed until ART-induced immune reconstitution elicits an exaggerated presentation of the disease [35,36]. Unmasking TB-IRIS usually occurs within three months of starting ART with high levels of clinical manifestations including lymphadenitis, abscess, and respiratory failure [37] (please see Figure 1).

Because there are no concrete laboratory-based biomarkers to confirm paradoxical TB-IRIS, the diagnosis is carried out mostly based on characteristic clinical presentations. While the AIDS Clinical Trials Group (ACTG) definition and the International Maternal Pediatric Adolescent AIDS Clinical Trials (IMPAACT) trial definition are employed in research settings, the International Network for the Study of HIV-associated IRIS (INSHI) consensus case definition is well validated and commonly used by researchers and clinicians to diagnose TB-IRIS [12,38]. This case definition requires a TB diagnosis with an initial adverse response to treatment, characteristic clinical symptoms and the exclusion of alternative causes of clinical deterioration including drug resistance, non-adherence, drug toxicity, and other new OIs [39,40]. At least one of the following major and two of the minor symptoms confirm the diagnosis of paradoxical TB-IRIS. The major symptoms include: (i) new or enlarging lymph nodes, cold abscess, or other focal tissue involvement, (ii) new or worsening radiological features of TB, (iii) new or worsening central nervous system TB and, (iv) new or worsening serositis. The minor symptoms include: (i) new or worsening constitutional symptoms, (ii) new or worsening respiratory symptoms, (iii) new or worsening abdominal pain and peritonitis, or hepatomegaly, splenomegaly, or abdominal adenopathy [11]. A recent prospective trial evaluating the diagnostic accuracy of the INSHI case definition in identifying paradoxical TB-IRIS revealed a sensitivity of 0.77 and a specificity of 0.86. Interestingly, the sensitivity and specificity rose to 0.89 and 0.88, respectively, when the minor INSHI criteria were replaced with objective measures such as an elevation of C-reactive protein (CRP) levels and fever [41]. While the case definition for paradoxical TB-IRIS is extensively defined, validated and widely used, the definition of unmasking TB-IRIS remains uncertain due to a hazy delineation of the necessary inflammatory components [37].

## 3. Incidence and Risk Factors

Studies from Africa and India have documented rates of incidence of paradoxical TB-IRIS among the adult population of PLWH ranging from 7 to 54%, with higher incidence among those with low CD4^+^ T cell counts [14,17,27] (Table 1). An incidence of 6.7% was found in a prospective cohort study of South African children starting ART while receiving TB therapy, suggesting a significantly lower burden of paradoxical TB-IRIS [42]. Numerous studies including randomized clinical trials have highlighted the association of low baseline CD4^+^ T cell counts, a high viral load at the time of initiation of ART, a shorter interval between TB treatment and ART initiation, and a high mycobacterial burden with the increased risk of paradoxical TB-IRIS [14,34,36,43]. Findings from a randomized clinical trial showed that the paradoxical TB-IRIS was more frequent among PLWH with early ART initiation than those initiating later [44].

Unmasking TB-IRIS is observed in about 1 to 4% of patients starting ART [12,45,52]. However, owing to the unclear knowledge and definition, and lack of confirmatory biomarkers, diagnosis of unmasking TB-IRIS remains a challenge and the differentiation of unmasking TB-IRIS from the presentation of non-IRIS TB following ART is difficult [12]. As observed in paradoxical TB-IRIS, the risk factors of unmasking TB-IRIS also include low baseline CD4^+^ T cell counts and a high viral load. Diagnosing TB in HIV itself being challenging further complicates the diagnosis of unmasking TB-IRIS [53]. Patients suspected of being infected with TB who have not started any TB therapy are at increased risk of developing unmasking TB-IRIS [54]. Lymphadenopathy on a chest radiograph, anaemia, elevated levels of CRP and weight loss are some of the risk factors associated with the development of unmasking TB-IRIS [45].

## 4. Challenges in Predicting and/or Diagnosing TB-IRIS

The detection of TB-lipoarabinomannan (LAM), a TB cell wall glycoprotein, in urine specimens is helpful in risk stratification in resource-limited settings because LAM antigen positivity correlates with disseminated TB and low CD4^+^ T cell counts. Studies have demonstrated a positive association between pre-ART LAM positivity and TB-IRIS [55,56]. Various studies have evaluated the potential role of various inflammatory cytokines and acute-phase reactants as biomarkers of TB-IRIS and the findings, though encouraging, are not concrete enough to be considered clinically useful now. Elevated levels of CRP, IL-6, IL-18, IP-10, IL-33, IFN-γ, and sCD14 individually or in combination have been investigated extensively as predictors for paradoxical TB-IRIS [15,50,57,58,59,60]. A prospective observational study from Botswana evaluated 26 plasma biomarkers in baseline specimens and revealed an association between TB-IRIS and lower growth factors such as IL-3, Th1 cytokine responses and low levels of IL-17 [16]. A study that investigated the role of IFN-γ release assays in response to mycobacterial antigens as a diagnostic marker for TB-IRIS did not reveal any significant differences between those who developed TB-IRIS and those who did not [61]. A recent study suggests that CD4^+^ T cell activation markers can predict TB-IRIS, and a combination of CD4^+^ and CD8^+^ T cell markers can help in diagnosing TB-IRIS [62].

Due to the ambiguous progression of the presenting symptoms, and the possibility of multiple OIs becoming unmasked in the same patient and thereby complicating the matter, diagnosing unmasking TB-IRIS presents a greater challenge than paradoxical TB-IRIS [12,63]. A UK-based study has demonstrated an association between elevated levels of CRP in patients and unmasking TB-IRIS [45]. Another study on a cohort of PLWH from South Africa revealed a significantly higher natural killer (NK) cell activation status and elevated serum levels of CRP and IL-8 [64].

## 5. Newer Host Genetic Markers to Predict TB-IRIS

A study suggests the role of genetics in the development of TB-IRIS as evaluated by the single nucleotide polymorphisms (SNP) in inflammatory and other immune-related genes. Polymorphisms in IL-18, and TNF genes were associated with the risk of developing TB-IRIS [65]. An evaluation of the role of leukotriene A4 hydroxylase (LTA4H) polymorphism in TB-IRIS revealed an increased incidence of IRIS among the mutant LTA4H genotypes [66]. A recent study from Brazil has demonstrated the association of an increased risk of IRIS onset with carriage of HLA-B*41 allele and KIR2DS1^+^HLA-C2 pair [67]. The same group from Brazil investigated the relationship between the SNPs of inflammasome genes and the onset of TB-IRIS. The study findings reveal the association of a higher risk of IRIS with the CT genotype or carrier allele T in the AIM2 gene, thereby confirming the involvement of polymorphisms in genes belonging to the innate immunity in the onset of TB-IRIS [60]. A recent study evaluated several blood transcriptomic TB signatures as tools to predict TB-IRIS before starting ART and the diagnosis of IRIS among a HIV-infected pediatric cohort initiating ART, and they demonstrated a modest but significant prognostic and diagnostic profile [68]. In a pilot metabolomic study of TB-IRIS, Silva et al. found that pathways involving the participation of arachidonic acid, linoleic acid, and glycerophospholipid metabolism are relevant markers in patients who are at increased risk of developing TB-IRIS [69]. The potential of using micro RNAs (miRNAs) as biomarkers for TB has been well documented and currently a clinical trial is underway to evaluate the circulating miRNA pattern as a biomarker for the prediction and prognosis of TB-IRIS [70,71]. 

Considering the fact that there are relatively fewer data available on host genetic factors than other systemic inflammation biomarkers and that the whole pathogenesis mechanism is dynamic and intertwined, others have developed a composite score for predicting TB-IRIS. This scoring merges the relevant biomarkers and involves a prediction model with a reasonably good predictive performance [72].

## 6. Immunopathogenesis of Paradoxical TB-IRIS

### 6.1. Deranged Functional Restoration of Cellular Immune Responses

Considering the important role played by the adaptive immune system in the defense against both HIV and TB, the functional reconstitution of adaptive immunity in the HIV/TB coinfected population due to the reversal of the state of immunosuppression in the presence of microbial antigen is expected and serves a beneficial role in the defense in most individuals [3]. 

Several studies have demonstrated the increase in antigen-specific CD4^+^ and CD8^+^ T cells in IRIS patients when compared to a non-IRIS control group [73,74]. Elevated levels of pro-inflammatory cytokines and chemokines play a major role in orchestrating exaggerated inflammatory responses in TB-IRIS patients [75]. However, recent studies have pointed out that the quantitative restoration of TB-specific CD4^+^ T cells is not a prerequisite for the development of TB-IRIS [57,76]. IRIS being increasingly reported among the non-HIV infected population following a period of immunosuppression points instead towards the common denominator of immunosuppression and the presence of an antigen irrespective of CD4^+^ T cell levels. While the initiation of ART can lead to the restoration of various aspects of immune responses against TB, it is not a complete restoration. While the counts of CD4^+^ T cells increase upon ART initiation, the ratios of memory to naïve subsets are unbalanced. Thus, the functional restoration of the T cells is unbalanced and eventually leads to a pathologically disproportionate inflammatory response [77]. In an earlier study, we demonstrated that the PLWH who develop TB-IRIS had higher Th1 responses to *M. tuberculosis* antigens before they started ART and that these responses were augmented after the initiation of ART [15]. Several other studies have similarly evaluated the cellular immune responses by measuring the protein levels of IFN-γ and other Th1-related factors including IL-2 and TNF [78,79]. A large study showing that the TB-IRIS patients had a significantly higher proportion of TB-specific IFN-γ, IL-2 and TNF secreting CD4^+^ T cells than the controls, demonstrating the key role of Th1 immune responses in TB-IRIS [78]. Interestingly, while they showed high levels of polyfunctional CD4^+^ T cell responses and IL-6 levels among the TB-IRIS patients, the non-IRIS controls additionally had a similar increase in CD4^+^ T cell responses while the IL-6 levels were low upon ART initiation. This supports the notion that the outcomes in TB-infected PLWH starting ART depend on the interactions of cellular immunity with the other arms of immunity. 

### 6.2. Role of Innate Immunity

Studies have suggested a role of myeloid cells in the initial stages of the TB-IRIS pathogenesis [80,81]. Transcriptional profiling of peripheral CD14^+^CD16^+^ monocytes isolated from TB-IRIS patients and non-IRIS patients demonstrated a differential abundance of genes associated with the complement system and pattern recognition receptors in TB-IRIS cases. Interestingly, evaluation of the complement system in the pathogenesis of TB-IRIS showed levels of the C1q and C1-inhibitor of the classical pathway to be significantly high at the baseline and that the balance of their ratio varied significantly at two weeks after the initiation of ART aligning with the onset of TB-IRIS, which indicates a potential role for these factors [80,81]. Various studies have documented the increased levels of monocyte activation markers such as plasma sCD14 and sCD163 [58,82]. Furthermore, among the TB-IRIS patients, the frequency of CD14^++^CD16^−^ monocytes was significantly higher and they had a high expression of CD163, an activation marker known to be associated with pro-inflammatory marker levels [82]. 

The roles of NK cells and invariant NK (iNKT) cells have also been implicated in the pathogenesis of TB-IRIS. A study from the Cambodian cohort showed a significantly higher baseline NK cell degranulation capacity among the patients who developed TB-IRIS, suggesting that the NK-degranulation levels could be used to identify patients with a higher risk of TB-IRIS [83]. An interesting microarray-based study from South Africa implicates the granule exocytosis pathway in the pathophysiology of TB-IRIS. The study demonstrated an abundance of perforin and granzyme B transcripts. The study reported significantly higher proportions of iNKT cells in TB-IRIS patients attributed to the high levels of perforin observed [84]. The authors in their recent study had demonstrated an increased iNKT cell frequency being associated with the development of TB-IRIS and that these cells were observed to be a CD4^+^ CD8^−^ subset depleted and degranulated at the onset time of TB-IRIS [85].

Three innate signaling pathways—a Toll-like receptor (TLR), triggering receptors expressed on myeloid cells 1 (TREM-1), and interleukin-1 (IL-1)—were found to be dominant among patients with TB-IRIS in a longitudinal transcriptomic profiling investigation. The findings from the transcriptomic data also correlated with the elevated concentrations of plasma cytokines in the TB-IRIS patients [86]. Inflammasomes are known to be the significant mediators of immune activation leading to the activation of innate cytokines such as IL-18.

**Figure 2:**
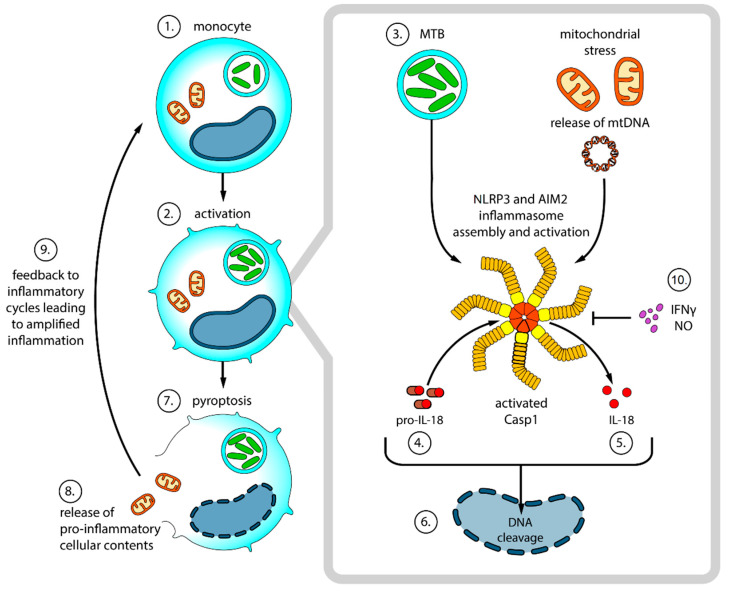
Proposed model of inflammasome-mediated pathogenesis of TB-IRIS. **(1–2**) In a microenvironment with low levels of IFN-γ, by interfering with the phagosome-lysosome fusion, MTB can evade the monocyte/macrophage bactericidal effectors [89] thereby continuing intracellular replication [90] leading to mitochondrial stress [91]. (**3**) Intracellular MTB accumulation and mtDNA result results inNLRP3 and AIM2 inflammasome assembly and activation of casp1. (**4–5**) In order to become active, the inactive form (pro-IL 18) of the potent pro-inflammatory cytokine IL-18 must first be processed by casp1 (**6–9**) In addition to processing IL-18, casp1 also promotes pyroptosis, an inflammatory form of programmed cell death. Pyroptosis results in the expulsion of mtDNA into the bloodstream which in turn amplifies inflammatory responses. (**10**) IFN-γ and NO negatively regulate NLRP3 and AIM2.

We had earlier demonstrated the association of elevated activated caspase-1 (casp1) in monocytes before ART initiation with the development of TB-IRIS. Seeing that inflammasomes are the activators of casp1, we investigated the relationship between the markers of inflammasome activation and the development of TB-IRIS and found that TB-IRIS was associated with the upregulation of genes encoding components of inflammasomes such as the nucleotide-binding oligomerization domain-like receptor family pyrin domain containing 3 (NLRP3) and absent in melanoma 2 (AIM2). Based on the findings, we documented the fact that the activity of inflammasome was over-activated in patients developing TB-IRIS upon ART initiation. We also demonstrated a correlation between high percentages of terminally differentiated NK cells and elevated IL-18Ra expression on CD4^+^ T cells and NK cells with the onset of TB-IRIS [87]. Figure 2 shows the proposed model of inflammasome-mediated pathogenesis of TB-IRIS. IL-18 has been suggested as the potential biomarker for predicting TB-IRIS, which highlights the important role of inflammasomes such as NLRP3 and AIM2 in the pathogenesis of TB-IRIS [57,87,88]. A recent study found, when investigating the distribution of SNPs of inflammasome genes, the proinflammatory cytokine profiles and their impact on the onset of TB-IRIS and an association between the C/T genotype and carrier-T in the AIM2 polymorphism with the increased risk of TB-IRIS [60].

A study involving a cohort of PLWH from South Africa demonstrated an association of TBM-IRIS with high neutrophil counts in cerebrospinal fluid (CSF) and increased expression of neutrophil mediators suggesting a role of neutrophils in the pathogenesis [92,93].

IL-1 activated by the inflammasome is known to induce neutrophil chemotaxis leading to infiltration at the site of TB-IRIS. Studies have demonstrated increased blood levels of neutrophils, human neutrophil peptides, and upregulated neutrophil activation gene transcripts being associated with TB-IRIS, thereby underscoring the importance of neutrophils in the pathogenesis of TB-IRIS [88,94,95]. Furthermore, in TB-IRIS patients there have been reports of elevated levels of matrix metalloproteinases (MMP) and endopeptidases and studies have shown that neutrophils drive the MMP section, which also highlights the significant role of neutrophils [16,56,93,96].

### 6.3. Role of Adaptive Immune Activation

Although the immunopathogenesis of TB-IRIS is mainly centered on the adaptive immune responses, characterized by the functional expansion of TB-specific T-cells ultimately leading to hypercytokinaemia, they contribute to amplifying the early pathogenesis caused by the innate immune arm as discussed earlier [3]. 

In one of our earlier studies, we demonstrated increased proportions of CD4^+^ and CD3^+^CD4^−^ T-cells reactive with TB antigens in PLWH who developed TB-IRIS during the first six weeks of ART initiation. We also found increased levels of IFN-γ and CXCL10 in supernatants of peripheral blood mononuclear cell (PBMC) cultures stimulated with TB-specific antigens in TB-IRIS patients. Our findings restate that the PLWH who develop TB-IRIS have increased Th1 responses to TB antigens before they start ART and that these responses are augmented upon the initiation of ART [15].

Based on the frequent occurrence of TB-IRIS among lymphopaenic patients upon starting ART, lymphopenia-induced T-cell homeostatic mechanisms and altered regulatory mechanisms are implicated in the immunopathogenesis of TB-IRIS. A study testing this hypothesis reported a predominance of highly activated PD-1^+^ HLA-DR^+^ and Ki67^+^ CD4^+^ T cells predominance before and during TB-IRIS among the patients who developed TB-IRIS [73]. Although a few studies implicated the role of impaired regulatory T-cells in uncontrolled inflammatory responses, several studies have documented increases in the number of Treg cells with reduced functionality in TB-IRIS patients [73,97,98,99,100]. Interestingly, there are conflicting reports on the kinetics of the number of Treg cells upon reconstitution, thereby calling for further detailed studies to explore the role of Treg cells in TB-IRIS [75,100].

A recent study analyzing the phenotypic landscape of T cells in PLWH with active TB initiating ART confirmed that TB-IRIS patients demonstrated prominent CD4^+^ lymphopenia before ART initiation. They also showed that among TB-IRIS patients there was increased T-cell activation, proliferation and cytotoxicity induced by ART [62]. 

Leukocyte chemotaxis is considered to play a role in the immunopathogenesis of TB-IRIS based on evidence from other forms of IRIS such as progressive multifocal leukoencephalopathy (PML) lesions IRIS. These PML lesions contained high percentages of CCR5^+^ cells, suggesting that chemokine receptors are involved in lymphocyte migration and the ensuing pathology [101]. Interestingly, we also demonstrated an increased proportion of CCR5^+^CD4^+^ T cells in TB-IRIS patients when compared to the non-TB-IRIS group [15]. A study from South India has shown that differential expression of CXCR3 and CCR6 on effector and memory CD4^+^ T cells was associated with TB-IRIS development in PLWH initiating ART. Expansion and functional restoration of central memory CD4^+^ T cells and corresponding cytokines after ART initiation in TB-IRIS patients highlight the arterial role of CD4^+^ T cell subsets in the immunopathogenesis of TB-IRIS [102]. A recent study from India revealed increased frequencies of antigen-experienced CD8^+^ T cells among TB-IRIS patients correlating with the AFB smear gradings. The study also demonstrates an inverse association between IRIS and naïve CXCR3^+^ CD8^+^ T cells substantiating the role of CD8^+^ T cells in TB-IRIS [103].

Thus, the immunopathogenesis of TB-IRIS involves a synergistic coupling of innate and adaptive arms of the immune system. Several predisposition factors for IRIS identified such as the low baseline CD4^+^ T cell count can tip the balance towards unwanted negative effects of immune reconstitution upon initiation of ART by PLWH with active TB. Inflammasome activation, the release of antigenic contents and stimulation of TLR could promote TB-specific T-cell proliferation, the generation of inflammatory cytokines and chemokines, as well as the recruitment of more immune cells to the site of inflammation. Hypercytokinaemia and inflammation could cause tissue damage further augmented by MMP release and matrix degradation. Host genetics also play a major role in these pathological processes.

## 7. Unmasking TB-IRIS

The immune mechanisms underlying unmasking TB-IRIS are similar to that of paradoxical TB-IRIS wherein exaggerated inflammatory immune responses occur following a rapid immune restoration [104]. A study on unmasking TB-IRIS demonstrated a distorted balance of T-cell phenotypes and an expansion of TB-specific Th1 responses only upon resolution of IRIS symptoms, indicating that the associated immunopathology differs from that of paradoxical TB-IRIS. The study also measured the thymic output based on the secretion of thymulin and found that it was compromised in patients with unmasking TB-IRIS [105]. Autopsy findings in a fatal case of unmasking TB-IRIS showed significant macrophage-dominated pulmonary infiltrate, thereby indicating the role of macrophages in the pathogenesis [106].

A study analyzing the transcriptomic profile of tuberculin skin test (TST) biopsies of unmasking TB-IRIS patients showed that even after recovery of CD4^+^ T cell counts these exaggerated immune responses persisted and were characterized by reduced IL-10 levels. The study also found association of Th2 responses with an increased transcriptional expression of interferon regulatory factor 4 (IRF4) [107]. The role of innate immune responses in unmasking TB-IRIS has also been implicated based on the increased activation of NK cells and the fact that elevated levels of CRP and IL-8 in plasma were observed in unmasking TB-IRIS patients [64]. Further detailed immunological studies are required to elucidate the immune mechanisms behind unmasking TB-IRIS to predict, diagnose and effectively manage this condition. 

## 8. Prevention and Management of TB-IRIS

A clinical trial investigating the effects of low-dose prednisone as a prophylaxis for people at risk showed a lower incidence of paradoxical TB-IRIS than placebo and no increased risk of other OIs and cancers were observed [108]. Statins are considered to be potential candidates for prophylactic management of TB-IRIS based on animal model studies and they are commonly prescribed to PLWH to lower their cholesterol levels, and are not associated with adverse effects [109,110]. The time of ART commencement is a critical risk factor for the development and severity of paradoxical risk in TB patients with HIV, with studies revealing a larger than two-fold increased risk of TB-IRIS in cases of early ART initiation [111,112,113].

For the management of paradoxical TB-IRIS, corticosteroids remain the mainstay first-line therapy with prednisone on a tapered dose over four weeks. A randomized clinical trial from South Africa demonstrated that the administration of prednisone improved the symptoms and quality-of-life indicators while reducing the requirements for prolonged hospital admissions [114]. Other alternatives such as nonsteroidal anti-inflammatory drugs and immunomodulators such as thalidomide, TNF-α inhibitors, and IL-6 blockers have been used for treatment and been reported on sporadically but have yet to be validated in randomized clinical trials [12].

For PLWH initiating ART, prophylactic TB treatment for PLWH initiating ART with trimethoprim-sulfamethoxazole and isoniazid/pyridoxine is recommended by the WHO [115]. This prophylaxis aims at treating latent TB and thereby preventing the reactivation of TB among those with severe immunosuppression [116]. Evidence from a multi-site clinical trial in Africa has demonstrated lesser unmasking TB-IRIS events and deaths in PLWH on this combined prophylactic therapy as compared with standard prophylaxis [117].

Treatment strategies are not well developed and evaluated for unmasked TB-IRIS. In severe cases of unmasking TB-IRIS, while earlier studies recommended withdrawal, a recent study suggested continuing the ART [11,118].

## 9. COVID-19 and TB-IRIS

In addition to the colossal impact on global public health and the global economy, both TB and COVID-19 share several similarities including the route of transmission. To make matters worse, there were several cases of COVID-19 and TB coinfection and limited access to TB care due to mitigation strategies devised for COVID-19 that led to increased TB deaths [119,120,121].

About 20% of the patients infected with SARS-CoV-2 can present as severe viral pneumonia progressing to acute respiratory distress syndrome with critical outcomes [122,123]. It has been evident that hyperinflammation caused by SARS-CoV-2-induced immunopathology, characterized by elevated levels of pro-inflammatory cytokines, lymphopaenia and neutrophilia leads to these clinical manifestations [124]. Although severe COVID-19 is a complication associated with SARS-CoV-2 infection, there remain features such as the exaggerated inflammatory responses and the immunopathology in infected tissues that thereby draw a similarity between its presentation and that of TB-IRIS. Interestingly, the similarities between COVID-19 and TB-IRIS go on to include the number of days to the onset of clinical manifestations, biomarkers linked to a higher risk of disease and death, elevated plasma levels of cytokines and chemokines and the interplay of innate and adaptive immune mechanisms [125]. Similar cytokine profiles have been observed in patients with severe COVID-19 and IRIS, pointing towards related pathophysiology and therapeutic options [126].

Treatment strategies in both these conditions focus mainly on the suppression of inflammatory responses. In the prevention and treatment of TB-IRIS and managing COVID-19, corticosteroid therapy has been observed to be only partially effective [108,127]. While treatment strategies targeting pro-inflammatory cytokines such as IL-6 were studied to be effective in TB-IRIS patients, there is no evidence of IL-6 inhibitors being effective against severe COVID-19 [3,128]. Alternatively, anti-inflammatory treatment for TB-IRIS as well as severe COVID-19 could evaluate the possible role of IL-18 inhibition based on the central role of IL-18 in the immunopathogenesis of both these conditions and preliminary findings [125,129]. Initiation of ART in patients coinfected with HIV and COVID-19 could pose a risk of developing IRIS and worsening outcomes [130]. It is interesting to note that just like TB in HIV patients, COVID-19 in immunocompromised patients can also lead to IRIS presentations [131,132,133].

## 10. Future Directions and Summary

In HIV/TB coinfected patients starting ART, TB-IRIS continues to be a source of morbidity and mortality. Although we now have significant insights into the immunopathogenesis, a clear understanding of the underlying mechanisms and identification of a sensitive and specific diagnostic markers are still pending. This calls for a multi-omic approach exploiting the recent technologies to better elucidate the immune mechanisms. Further structured clinical trials are required to optimize the prevention and management strategies for better outcomes. 

## Figures and Tables

**Table 1:** Frequency of occurrence of TB-IRIS among HIV/TB co-infected population in studies from different countries.

Author and Year of Publication	Country	Frequency (%)
Haddow, L.J., 2012 [45]	South Africa	13.7
Achenbach. C.J., 2012 [46]	USA	16
Limmahakhun, S., 2012 [47]	Thailand	6.1
Worodria, W., 2012 [48]	Uganda	20.9
Ali, K., 2012 [49]	Ethiopia	22.4
Narendran, G., 2013 [50]	India	54.2
Tan, H.Y., 2014 [20]	Malaysia	9.4
Xue, M., 2020 [51]	China	22.6

## References

[1] Global Tuberculosis Report 2021. https://www.who.int/publications-detail-redirect/9789240037021.

[2] Shankar E.M., Vignesh R., Ellegård R., Barathan M., Chong Y.K., Bador M.K., Rukumani D.V., Sabet N.S., Kamarulzaman A., Velu V. (2014). HIV-Mycobacterium Tuberculosis Co-Infection: A “danger-Couple Model” of Disease Pathogenesis. Pathog. Dis..

[3] Cevaal P.M., Bekker L.-G., Hermans S. (2019). TB-IRIS Pathogenesis and New Strategies for Intervention: Insights from Related Inflammatory Disorders. Tuberculosis.

[4] Kwan C.K., Ernst J.D. (2011). HIV and Tuberculosis: A Deadly Human Syndemic. Clin. Microbiol. Rev..

[5] Waters R., Ndengane M., Abrahams M.-R., Diedrich C.R., Wilkinson R.J., Coussens A.K. (2020). The Mtb-HIV Syndemic Interaction: Why Treating M. Tuberculosis Infection May Be Crucial for HIV-1 Eradication. Future Virol..

[6] Wong K., Nguyen J., Blair L., Banjanin M., Grewal B., Bowman S., Boyd H., Gerstner G., Cho H.J., Panfilov D. (2020). Pathogenesis of Human Immunodeficiency Virus-Mycobacterium Tuberculosis Co-Infection. J. Clin. Med..

[7] Tuberculosis (TB). https://www.who.int/news-room/fact-sheets/detail/tuberculosis.

[8] Sharan R., Bucşan A.N., Ganatra S., Paiardini M., Mohan M., Mehra S., Khader S., Kaushal D. (2020). Chronic Immune Activation in TB/HIV Co-Infection. Trends Microbiol..

[9] Dupont M., Souriant S., Balboa L., Vu Manh T.-P., Pingris K., Rousset S., Cougoule C., Rombouts Y., Poincloux R., Ben Neji M. (2020). Tuberculosis-Associated IFN-I Induces Siglec-1 on Tunneling Nanotubes and Favors HIV-1 Spread in Macrophages. Elife.

[10] Suthar A.B., Lawn S.D., del Amo J., Getahun H., Dye C., Sculier D., Sterling T.R., Chaisson R.E., Williams B.G., Harries A.D. (2012). Antiretroviral Therapy for Prevention of Tuberculosis in Adults with HIV: A Systematic Review and Meta-Analysis. PLoS Med..

[11] Walker N.F., Stek C., Wasserman S., Wilkinson R.J., Meintjes G. (2018). The Tuberculosis-Associated Immune Reconstitution Inflammatory Syndrome: Recent Advances in Clinical and Pathogenesis Research. Curr. Opin. HIV AIDS.

[12] Quinn C.M., Poplin V., Kasibante J., Yuquimpo K., Gakuru J., Cresswell F.V., Bahr N.C. (2020). Tuberculosis IRIS: Pathogenesis, Presentation, and Management across the Spectrum of Disease. Life.

[13] Shankar E.M., Vignesh R., Murugavel K.G., Balakrishnan P., Sekar R., Lloyd C.A., Solomon S., Kumarasamy N. (2007). Immune Reconstitution Inflammatory Syndrome in Association with HIV/AIDS and Tuberculosis: Views over Hidden Possibilities. AIDS Res. Ther..

[14] Vignesh R., Swathirajan C.R., Solomon S.S., Shankar E.M., Murugavel K.G. (2017). Risk Factors and Frequency of Tuberculosis-Associated Immune Reconstitution Inflammatory Syndrome among HIV/Tuberculosis Co-Infected Patients in Southern India. Indian J. Med. Microbiol..

[15] Vignesh R., Kumarasamy N., Lim A., Solomon S., Murugavel K.G., Balakrishnan P., Solomon S.S., Mayer K.H., Swathirajan C.R., Chandrasekaran E. (2013). TB-IRIS after Initiation of Antiretroviral Therapy Is Associated with Expansion of Preexistent Th1 Responses against Mycobacterium Tuberculosis Antigens. JAIDS J. Acquir. Immune Defic. Syndr..

[16] Ravimohan S., Tamuhla N., Steenhoff A.P., Letlhogile R., Nfanyana K., Bellamy S.L., MacGregor R.R., Gross R., Weissman D., Bisson G.P. (2015). Immunological Profiling of Tuberculosis-Associated Immune Reconstitution Inflammatory Syndrome and Non-Immune Reconstitution Inflammatory Syndrome Death in HIV-Infected Adults with Pulmonary Tuberculosis Starting Antiretroviral Therapy: A Prospective Observational Cohort Study. Lancet Infect. Dis..

[17] Gopalan N., Santhanakrishnan R.K., Palaniappan A.N., Menon P.A., Lakshman S., Chandrasekaran P., Sivaramakrishnan G.N., Reddy D., Kannabiran B.P., Agiboth H.K.K. (2018). Daily vs Intermittent Antituberculosis Therapy for Pulmonary Tuberculosis in Patients With HIV: A Randomized Clinical Trial. JAMA Intern. Med..

[18] Li L., Li J., Chai C., Liu T., Li P., Qu M., Zhao H. (2021). Association of CD4 T Cell Count and Optimal Timing of Antiretroviral Therapy Initiation with Immune Reconstitution Inflammatory Syndrome and All-Cause Mortality for HIV-Infected Adults with Newly Diagnosed Pulmonary Tuberculosis: A Systematic Review and Meta-Analysis. Int. J. Clin. Exp. Pathol..

[19] Tan D.B.A., Yong Y.K., Tan H.Y., Kamarulzaman A., Tan L.H., Lim A., James I., French M., Price P. (2008). Immunological Profiles of Immune Restoration Disease Presenting as Mycobacterial Lymphadenitis and Cryptococcal Meningitis. HIV Med..

[20] Tan H.Y., Yong Y.K., Lim S.H., Ponnampalavanar S., Omar S.F.S., Pang Y.K., Kamarulzaman A., Price P., Crowe S.M., French M.A. (2014). Tuberculosis (TB)-Associated Immune Reconstitution Inflammatory Syndrome in TB-HIV Co-Infected Patients in Malaysia: Prevalence, Risk Factors, and Treatment Outcomes. Sex Health.

[21] Yapa H.M., Kim H.-Y., Petoumenos K., Post F.A., Jiamsakul A., De Neve J.-W., Tanser F., Iwuji C., Baisley K., Shahmanesh M. (2021). CD4+ T-Cell Count at Antiretroviral Therapy Initiation in the “Treat-All” Era in Rural South Africa: An Interrupted Time Series Analysis. Clin. Infect. Dis..

[22] Ismail S.D., Pankrac J., Ndashimye E., Prodger J.L., Abrahams M.-R., Mann J.F.S., Redd A.D., Arts E.J. (2021). Addressing an HIV Cure in LMIC. Retrovirology.

[23] Carmona S., Bor J., Nattey C., Maughan-Brown B., Maskew M., Fox M.P., Glencross D.K., Ford N., MacLeod W.B. (2018). Persistent High Burden of Advanced HIV Disease Among Patients Seeking Care in South Africa’s National HIV Program: Data From a Nationwide Laboratory Cohort. Clin. Infect. Dis..

[24] Bock P., Fatti G., Ford N., Jennings K., Kruger J., Gunst C., Louis F., Grobbelaar N., Shanaube K., Floyd S. (2018). Attrition When Providing Antiretroviral Treatment at CD4 Counts >500 cells/ΜL at Three Government Clinics Included in the HPTN 071 (PopART) Trial in South Africa. PLoS ONE.

[25] Lebelonyane R., Bachanas P., Block L., Ussery F., Abrams W., Roland M., Theu J., Kapanda M., Matambo S., Lockman S. (2020). Rapid Antiretroviral Therapy Initiation in the Botswana Combination Prevention Project: A Quasi-Experimental before and after Study. Lancet HIV.

[26] Colocci I., Perlo J., Rajagopal S.S., Betancourt T.S., Pradeep A., Mayer K.H., Kumarasamy N., O’Cleirigh C., Katz I.T., Chan B.T. (2021). Economic Vulnerability and Non-Initiation of Antiretroviral Therapy in India: A Qualitative Study. AIDS Care.

[27] Ravimohan S., Tamuhla N., Kung S.-J., Nfanyana K., Steenhoff A.P., Gross R., Weissman D., Bisson G.P. (2016). Matrix Metalloproteinases in Tuberculosis-Immune Reconstitution Inflammatory Syndrome and Impaired Lung Function Among Advanced HIV/TB Co-Infected Patients Initiating Antiretroviral Therapy. EBioMedicine.

[28] Narendran G., Jyotheeswaran K., Senguttuvan T., Vinhaes C.L., Santhanakrishnan R.K., Manoharan T., Selvaraj A., Chandrasekaran P., Menon P.A., Bhavani K.P. (2020). Characteristics of Paradoxical Tuberculosis-Associated Immune Reconstitution Inflammatory Syndrome and Its Influence on Tuberculosis Treatment Outcomes in Persons Living with HIV. Int. J. Infect. Dis..

[29] Sharma S.K., Soneja M. (2011). HIV & Immune Reconstitution Inflammatory Syndrome (IRIS). Indian J. Med. Res..

[30] Manzardo C., Guardo A.C., Letang E., Plana M., Gatell J.M., Miro J.M. (2015). Opportunistic Infections and Immune Reconstitution Inflammatory Syndrome in HIV-1-Infected Adults in the Combined Antiretroviral Therapy Era: A Comprehensive Review. Expert Rev. Anti. Infect. Ther..

[31] Nelson A.M., Manabe Y.C., Lucas S.B. (2017). Immune Reconstitution Inflammatory Syndrome (IRIS): What Pathologists Should Know. Semin. Diagn. Pathol..

[32] Rewari B.B., Kumar A., Mandal P.P., Puri A.K. (2021). HIV TB Coinfection—Perspectives from India. Expert. Rev. Respir. Med..

[33] Manosuthi W., Van Tieu H., Mankatitham W., Lueangniyomkul A., Ananworanich J., Avihingsanon A., Siangphoe U., Klongugkara S., Likanonsakul S., Thawornwan U. (2009). Clinical Case Definition and Manifestations of Paradoxical Tuberculosis-Associated Immune Reconstitution Inflammatory Syndrome. AIDS.

[34] Namale P.E., Abdullahi L.H., Fine S., Kamkuemah M., Wilkinson R.J., Meintjes G. (2015). Paradoxical TB-IRIS in HIV-Infected Adults: A Systematic Review and Meta-Analysis. Future Microbiol..

[35] Zhao Y., Hohlfeld A., Namale P., Meintjes G., Maartens G., Engel M.E. (2022). Risk of Immune Reconstitution Inflammatory Syndrome with Integrase Inhibitors versus Other Classes of Antiretrovirals: A Systematic Review and Meta-Analysis of Randomised Trials. J. Acquir. Immune Defic. Syndr..

[36] Manosuthi W., Wiboonchutikul S., Sungkanuparph S. (2016). Integrated Therapy for HIV and Tuberculosis. AIDS Res. Ther..

[37] Meintjes G., Lawn S.D., Scano F., Maartens G., French M.A., Worodria W., Elliott J.H., Murdoch D., Wilkinson R.J., Seyler C. (2008). Tuberculosis-Associated Immune Reconstitution Inflammatory Syndrome: Case Definitions for Use in Resource-Limited Settings. Lancet Infect. Dis..

[38] Bell L.C.K., Breen R., Miller R.F., Noursadeghi M., Lipman M. (2015). Paradoxical Reactions and Immune Reconstitution Inflammatory Syndrome in Tuberculosis. Int. J. Infect. Dis..

[39] Eshun-Wilson I., Havers F., Nachega J.B., Prozesky H.W., Taljaard J.J., Zeier M.D., Cotton M., Simon G., Soentjens P. (2010). Evaluation of Paradoxical TB-Associated IRIS with the Use of Standardized Case Definitions for Resource-Limited Settings. J. Int. Assoc. Physicians AIDS Care (Chic).

[40] Haddow L.J., Moosa M.-Y.S., Easterbrook P.J. (2010). Validation of a Published Case Definition for Tuberculosis-Associated Immune Reconstitution Inflammatory Syndrome. AIDS.

[41] Stek C., Buyze J., Menten J., Schutz C., Thienemann F., Blumenthal L., Maartens G., Boyles T., Wilkinson R.J., Meintjes G. (2021). Diagnostic Accuracy of the INSHI Consensus Case Definition for the Diagnosis of Paradoxical Tuberculosis-IRIS. J. Acquir. Immune. Defic. Syndr..

[42] Van Rie A., Sawry S., Link-Gelles R., Madhi S., Fairlie L., Verwey C., Mahomed N., Murdoch D., Moultrie H. (2016). Paradoxical Tuberculosis-Associated Immune Reconstitution Inflammatory Syndrome in Children. Pediatr. Pulmonol..

[43] Luetkemeyer A.F., Kendall M.A., Nyirenda M., Wu X., Ive P., Benson C.A., Andersen J.W., Swindells S., Sanne I.M., Havlir D.V. (2014). Tuberculosis Immune Reconstitution Inflammatory Syndrome in A5221 STRIDE: Timing, Severity, and Implications for HIV-TB Programs. J. Acquir. Immune. Defic. Syndr..

[44] Amogne W., Aderaye G., Habtewold A., Yimer G., Makonnen E., Worku A., Sonnerborg A., Aklillu E., Lindquist L. (2015). Efficacy and Safety of Antiretroviral Therapy Initiated One Week after Tuberculosis Therapy in Patients with CD4 Counts < 200 Cells/ΜL: TB-HAART Study, a Randomized Clinical Trial. PLoS ONE.

[45] Haddow L.J., Moosa M.-Y.S., Mosam A., Moodley P., Parboosing R., Easterbrook P.J. (2012). Incidence, Clinical Spectrum, Risk Factors and Impact of HIV-Associated Immune Reconstitution Inflammatory Syndrome in South Africa. PLoS ONE.

[46] Achenbach C.J., Harrington R.D., Dhanireddy S., Crane H.M., Casper C., Kitahata M.M. (2012). Paradoxical Immune Reconstitution Inflammatory Syndrome in HIV-Infected Patients Treated With Combination Antiretroviral Therapy After AIDS-Defining Opportunistic Infection. Clin. Infect. Dis..

[47] Limmahakhun S., Chaiwarith R., Nuntachit N., Sirisanthana T., Supparatpinyo K. (2012). Treatment Outcomes of Patients Co-Infected with Tuberculosis and HIV at Chiang Mai University Hospital, Thailand. Int. J. STD AIDS.

[48] Worodria W., Menten J., Massinga-Loembe M., Mazakpwe D., Bagenda D., Koole O., Mayanja-Kizza H., Kestens L., Mugerwa R., Reiss P. (2012). Clinical Spectrum, Risk Factors and Outcome of Immune Reconstitution Inflammatory Syndrome in Patients with Tuberculosis-HIV Coinfection. Antivir. Ther..

[49] Ali K., Klotz S.A. (2012). The Immune Reconstitution Inflammatory Syndrome with Tuberculosis: A Common Problem in Ethiopian HIV-Infected Patients Beginning Antiretroviral Therapy. J. Int. Assoc. Physicians AIDS Care (Chic).

[50] Narendran G., Andrade B.B., Porter B.O., Chandrasekhar C., Venkatesan P., Menon P.A., Subramanian S., Anbalagan S., Bhavani K.P., Sekar S. (2013). Paradoxical Tuberculosis Immune Reconstitution Inflammatory Syndrome (TB-IRIS) in HIV Patients with Culture Confirmed Pulmonary Tuberculosis in India and the Potential Role of IL-6 in Prediction. PLoS ONE.

[51] Xue M., Xie R., Pang Y., Yan S., Du Y., Guan C., Chen B. (2020). Prevalence and Risk Factors of Paradoxical Tuberculosis Associated Immune Reconstitution Inflammatory Syndrome among HIV-Infected Patients in Beijing, China. BMC Infect. Dis..

[52] Worodria W., Massinga-Loembe M., Mayanja-Kizza H., Namaganda J., Kambugu A., Manabe Y.C., Kestens L., Colebunders R. (2011). Antiretroviral Treatment-Associated Tuberculosis in a Prospective Cohort of HIV-Infected Patients Starting ART. Clin. Dev. Immunol..

[53] Méndez-Samperio P. (2017). Diagnosis of Tuberculosis in HIV Co-Infected Individuals: Current Status, Challenges and Opportunities for the Future. Scand. J. Immunol..

[54] Pathmanathan I., Dokubo E.K., Shiraishi R.W., Agolory S.G., Auld A.F., Onotu D., Odafe S., Dalhatu I., Abiri O., Debem H.C. (2017). Incidence and Predictors of Tuberculosis among HIV-Infected Adults after Initiation of Antiretroviral Therapy in Nigeria, 2004–2012. PLoS ONE.

[55] Bulterys M.A., Wagner B., Redard-Jacot M., Suresh A., Pollock N.R., Moreau E., Denkinger C.M., Drain P.K., Broger T. (2019). Point-Of-Care Urine LAM Tests for Tuberculosis Diagnosis: A Status Update. J. Clin. Med..

[56] Walker N.F., Wilkinson K.A., Meintjes G., Tezera L.B., Goliath R., Peyper J.M., Tadokera R., Opondo C., Coussens A.K., Wilkinson R.J. (2017). Matrix Degradation in Human Immunodeficiency Virus Type 1–Associated Tuberculosis and Tuberculosis Immune Reconstitution Inflammatory Syndrome: A Prospective Observational Study. Clin. Infect. Dis..

[57] Tan H.Y., Yong Y.K., Andrade B.B., Shankar E.M., Ponnampalavanar S., Omar S.F.S., Narendran G., Kamarulzaman A., Swaminathan S., Sereti I. (2015). Plasma Interleukin-18 Levels Are a Biomarker of Innate Immune Responses That Predict and Characterize Tuberculosis-Associated Immune Reconstitution Inflammatory Syndrome. AIDS.

[58] Musselwhite L.W., Andrade B.B., Ellenberg S.S., Tierney A., Belaunzaran-Zamudio P.F., Rupert A., Lederman M.M., Sanne I., Sierra Madero J.G., Sereti I. (2016). Vitamin D, D-Dimer, Interferon γ, and SCD14 Levels Are Independently Associated with Immune Reconstitution Inflammatory Syndrome: A Prospective, International Study. EBioMedicine.

[59] Oliver B.G., Elliott J.H., Price P., Phillips M., Saphonn V., Vun M.C., Kaldor J.M., Cooper D.A., French M.A. (2010). Mediators of Innate and Adaptive Immune Responses Differentially Affect Immune Restoration Disease Associated with Mycobacterium Tuberculosis in HIV Patients Beginning Antiretroviral Therapy. J. Infect. Dis..

[60] de Sá N.B.R., de Souza N.C.S., Neira-Goulart M., Ribeiro-Alves M., Da Silva T.P., Pilotto J.H., Rolla V.C., Giacoia-Gripp C.B.W., de Oliveira Pinto L.M., Scott-Algara D. (2022). Inflammasome Genetic Variants Are Associated with Tuberculosis, HIV-1 Infection, and TB/HIV-Immune Reconstitution Inflammatory Syndrome Outcomes. Front. Cell Infect. Microbiol..

[61] Dirix V., Schepers K., Massinga-Loembe M., Worodria W., Colebunders R., Singh M., Locht C., Kestens L., Mascart F. (2016). TB-IRIS study group Added Value of Long-Term Cytokine Release Assays to Detect Mycobacterium Tuberculosis Infection in HIV-Infected Subjects in Uganda. J. Acquir. Immune. Defic. Syndr..

[62] Tibúrcio R., Barreto-Duarte B., Naredren G., Queiroz A.T.L., Anbalagan S., Nayak K., Ravichandran N., Subramani R., Antonelli L.R.V., Satagopan K. (2021). Dynamics of T-Lymphocyte Activation Related to Paradoxical Tuberculosis-Associated Immune Reconstitution Inflammatory Syndrome in Persons With Advanced HIV. Front. Immunol..

[63] Ellis J., Cresswell F.V., Rhein J., Ssebambulidde K., Boulware D.R. (2018). Cryptococcal Meningitis and Tuberculous Meningitis Co-Infection in HIV-Infected Ugandan Adults. Open Forum. Infect. Dis..

[64] Conradie F., Foulkes A.S., Ive P., Yin X., Roussos K., Glencross D.K., Lawrie D., Stevens W., Montaner L.J., Sanne I. (2011). Natural Killer Cell Activation Distinguishes M. Tuberculosis-Mediated Immune Reconstitution Syndrome (IRIS) from Chronic HIV and HIV-MTB Co-Infection. J. Acquir. Immune. Defic. Syndr..

[65] Affandi J.S., Kumar M., Agarwal U., Singh S., Price P. (2013). The Search for a Genetic Factor Associating with Immune Restoration Disease in HIV Patients Co-Infected with Mycobacterium Tuberculosis. Dis. Markers.

[66] Narendran G., Kavitha D., Karunaianantham R., Gil-Santana L., Almeida-Junior J.L., Reddy S.D., Kumar M.M., Hemalatha H., Jayanthi N.N., Ravichandran N. (2016). Role of LTA4H Polymorphism in Tuberculosis-Associated Immune Reconstitution Inflammatory Syndrome Occurrence and Clinical Severity in Patients Infected with HIV. PLoS ONE.

[67] de Sá N.B.R., Ribeiro-Alves M., da Silva T.P., Pilotto J.H., Rolla V.C., Giacoia-Gripp C.B.W., Scott-Algara D., Morgado M.G., Teixeira S.L.M. (2020). Clinical and Genetic Markers Associated with Tuberculosis, HIV-1 Infection, and TB/HIV-Immune Reconstitution Inflammatory Syndrome Outcomes. BMC Infect. Dis..

[68] Mbandi S.K., Painter H., Penn-Nicholson A., Toefy A., Erasmus M., Hanekom W.A., Scriba T.J., Lai R.P.J., Marais S., Fletcher H.A. (2022). Host Transcriptomic Signatures of Tuberculosis Can Predict Immune Reconstitution Inflammatory Syndrome in HIV Patients. Eur. J. Immunol..

[69] Silva C.A.M., Graham B., Webb K., Ashton L.V., Harton M., Luetkemeyer A.F., Bokatzian S., Almubarak R., Mahapatra S., Hovind L. (2019). A Pilot Metabolomics Study of Tuberculosis Immune Reconstitution Inflammatory Syndrome. Int. J. Infect. Dis..

[70] ANRS (2020). Emerging Infectious Diseases. Micro RNA as Prediction and/or Prognostic Markers of IRIS in TB-HIV Co-Infected Patients.

[71] Sabir N., Hussain T., Shah S.Z.A., Peramo A., Zhao D., Zhou X. (2018). MiRNAs in Tuberculosis: New Avenues for Diagnosis and Host-Directed Therapy. Front. Microbiol..

[72] Vinhaes C.L., Sheikh V., Oliveira-de-Souza D., Wang J., Rupert A., Roby G., Arriaga M.B., Fukutani K.F., Sawe F., Shaffer D. (2021). An Inflammatory Composite Score Predicts Mycobacterial Immune Reconstitution Inflammatory Syndrome in People with Advanced HIV: A Prospective International Cohort Study. J. Infect. Dis..

[73] Antonelli L.R.V., Mahnke Y., Hodge J.N., Porter B.O., Barber D.L., DerSimonian R., Greenwald J.H., Roby G., Mican J., Sher A. (2010). Elevated Frequencies of Highly Activated CD4+ T Cells in HIV+ Patients Developing Immune Reconstitution Inflammatory Syndrome. Blood.

[74] Espinosa E., Romero-Rodríguez D.P., Cantoral-Díaz M.-T., Reyes-Terán G. (2013). Transient Expansion of Activated CD8(+) T Cells Characterizes Tuberculosis-Associated Immune Reconstitution Inflammatory Syndrome in Patients with HIV: A Case Control Study. J. Inflamm. (Lond).

[75] Haridas V., Pean P., Jasenosky L.D., Madec Y., Laureillard D., Sok T., Sath S., Borand L., Marcy O., Chan S. (2015). TB-IRIS, T-Cell Activation, and Remodeling of the T-Cell Compartment in Highly Immunosuppressed HIV-Infected Patients with TB. AIDS.

[76] Walker N.F., Meintjes G., Wilkinson R.J. (2013). HIV-1 and the Immune Response to TB. Future Virol..

[77] Robbins G.K., Spritzler J.G., Chan E.S., Asmuth D.M., Gandhi R.T., Rodriguez B., Skowron G., Skolnik P.R., Shafer R.W., Pollard R.B. (2009). Incomplete Reconstitution of T Cell Subsets on Combination Antiretroviral Therapy in the AIDS Clinical Trials Group Protocol 384. Clin. Infect. Dis. Off. Publ. Infect. Dis. Soc. Am..

[78] Ravimohan S., Tamuhla N., Nfanyana K., Steenhoff A.P., Letlhogile R., Frank I., MacGregor R.R., Gross R., Weissman D., Bisson G.P. (2016). Robust Reconstitution of Tuberculosis-Specific Polyfunctional CD4+ T-Cell Responses and Rising Systemic Interleukin 6 in Paradoxical Tuberculosis-Associated Immune Reconstitution Inflammatory Syndrome. Clin. Infect. Dis..

[79] Ravimohan S., Tamuhla N., Steenhoff A.P., Letlhogile R., Makutu D.K., Nfanyana K., Rantleru T., Tierney A., Nkakana K., Schwartz A.B. (2013). Early Immunologic Failure Is Associated with Early Mortality among Advanced HIV-Infected Adults Initiating Antiretroviral Therapy with Active Tuberculosis. J. Infect. Dis..

[80] Tran H.T.T., Van den Bergh R., Vu T.N., Laukens K., Worodria W., Loembé M.M., Colebunders R., Kestens L., De Baetselier P., Raes G. (2014). The Role of Monocytes in the Development of Tuberculosis-Associated Immune Reconstitution Inflammatory Syndrome. Immunobiology.

[81] Tran H.T.T., Van den Bergh R., Loembé M.M., Worodria W., Mayanja-Kizza H., Colebunders R., Mascart F., Stordeur P., Kestens L., De Baetselier P. (2013). Modulation of the Complement System in Monocytes Contributes to Tuberculosis-Associated Immune Reconstitution Inflammatory Syndrome. AIDS.

[82] Andrade B.B., Singh A., Narendran G., Schechter M.E., Nayak K., Subramanian S., Anbalagan S., Jensen S.M.R., Porter B.O., Antonelli L.R. (2014). Mycobacterial Antigen Driven Activation of CD14++CD16- Monocytes Is a Predictor of Tuberculosis-Associated Immune Reconstitution Inflammatory Syndrome. PLoS Pathog..

[83] Pean P., Nerrienet E., Madec Y., Borand L., Laureillard D., Fernandez M., Marcy O., Sarin C., Phon K., Taylor S. (2012). Natural Killer Cell Degranulation Capacity Predicts Early Onset of the Immune Reconstitution Inflammatory Syndrome (IRIS) in HIV-Infected Patients with Tuberculosis. Blood.

[84] Wilkinson K.A., Walker N.F., Meintjes G., Deffur A., Nicol M.P., Skolimowska K.H., Matthews K., Tadokera R., Seldon R., Maartens G. (2015). Cytotoxic Mediators in Paradoxical HIV-Tuberculosis Immune Reconstitution Inflammatory Syndrome. J. Immunol..

[85] Walker N.F., Opondo C., Meintjes G., Jhilmeet N., Friedland J.S., Elkington P.T., Wilkinson R.J., Wilkinson K.A. (2020). Invariant Natural Killer T-Cell Dynamics in Human Immunodeficiency Virus-Associated Tuberculosis. Clin. Infect. Dis..

[86] Lai R.P.J., Meintjes G., Wilkinson K.A., Graham C.M., Marais S., Van der Plas H., Deffur A., Schutz C., Bloom C., Munagala I. (2015). HIV-Tuberculosis-Associated Immune Reconstitution Inflammatory Syndrome Is Characterized by Toll-like Receptor and Inflammasome Signalling. Nat. Commun..

[87] Tan H.Y., Yong Y.K., Shankar E.M., Paukovics G., Ellegård R., Larsson M., Kamarulzaman A., French M.A., Crowe S.M. (2016). Aberrant Inflammasome Activation Characterizes Tuberculosis-Associated Immune Reconstitution Inflammatory Syndrome. J. Immunol..

[88] Marais S., Lai R.P.J., Wilkinson K.A., Meintjes G., O’Garra A., Wilkinson R.J. (2017). Inflammasome Activation Underlying Central Nervous System Deterioration in HIV-Associated Tuberculosis. J. Infect. Dis..

[89] Herbst S., Schaible U.E., Schneider B.E. (2011). Interferon Gamma Activated Macrophages Kill Mycobacteria by Nitric Oxide Induced Apoptosis. PLoS ONE.

[90] de Chastellier C. (2009). The Many Niches and Strategies Used by Pathogenic Mycobacteria for Survival within Host Macrophages. Immunobiology.

[91] Jamwal S., Midha M.K., Verma H.N., Basu A., Rao K.V.S., Manivel V. (2013). Characterizing Virulence-Specific Perturbations in the Mitochondrial Function of Macrophages Infected with Mycobacterium Tuberculosis. Sci. Rep..

[92] Marais S., Meintjes G., Pepper D.J., Dodd L.E., Schutz C., Ismail Z., Wilkinson K.A., Wilkinson R.J. (2013). Frequency, Severity, and Prediction of Tuberculous Meningitis Immune Reconstitution Inflammatory Syndrome. Clin. Infect. Dis..

[93] Marais S., Wilkinson K.A., Lesosky M., Coussens A.K., Deffur A., Pepper D.J., Schutz C., Ismail Z., Meintjes G., Wilkinson R.J. (2014). Neutrophil-Associated Central Nervous System Inflammation in Tuberculous Meningitis Immune Reconstitution Inflammatory Syndrome. Clin. Infect. Dis..

[94] Nakiwala J.K., Walker N.F., Diedrich C.R., Worodria W., Meintjes G., Wilkinson R.J., Mayanja-Kizza H., Colebunders R., Kestens L., Wilkinson K.A. (2018). Neutrophil Activation and Enhanced Release of Granule Products in HIV-TB Immune Reconstitution Inflammatory Syndrome. J. Acquir. Immune. Defic. Syndr..

[95] Ma J., Zhao F., Su W., Li Q., Li J., Ji J., Deng Y., Zhou Y., Wang X., Yang H. (2018). Zinc Finger and Interferon-Stimulated Genes Play a Vital Role in TB-IRIS Following HAART in AIDS. Per. Med..

[96] Tadokera R., Meintjes G.A., Wilkinson K.A., Skolimowska K.H., Walker N., Friedland J.S., Maartens G., Elkington P.T.G., Wilkinson R.J. (2014). Matrix Metalloproteinases and Tissue Damage in HIV-Tuberculosis Immune Reconstitution Inflammatory Syndrome. Eur. J. Immunol..

[97] Shankar E.M., Vignesh R., Velu V., Murugavel K.G., Sekar R., Balakrishnan P., Lloyd C.A., Saravanan S., Solomon S., Kumarasamy N. (2008). Does CD4+ CD25+ Foxp3+ Cell (Treg) and IL-10 Profile Determine Susceptibility to Immune Reconstitution Inflammatory Syndrome (IRIS) in HIV Disease?. J. Inflamm..

[98] Gopalan N., Andrade B.B., Swaminathan S. (2014). Tuberculosis-Immune Reconstitution Inflammatory Syndrome in HIV: From Pathogenesis to Prediction. Expert. Rev. Clin. Immunol..

[99] Lai R.P.J., Meintjes G., Wilkinson R.J. (2016). HIV-1 Tuberculosis-Associated Immune Reconstitution Inflammatory Syndrome. Semin. Immunopathol..

[100] Zaidi I., Peterson K., Jeffries D., Whittle H., de Silva T., Rowland-Jones S., Jaye A., de Jong B.C. (2012). Immune Reconstitution Inflammatory Syndrome and the Influence of T Regulatory Cells: A Cohort Study in The Gambia. PLoS ONE.

[101] Martin-Blondel G., Bauer J., Uro-Coste E., Biotti D., Averseng-Peaureaux D., Fabre N., Dumas H., Bonneville F., Lassmann H., Marchou B. (2015). Therapeutic Use of CCR5 Antagonists Is Supported by Strong Expression of CCR5 on CD8(+) T Cells in Progressive Multifocal Leukoencephalopathy-Associated Immune Reconstitution Inflammatory Syndrome. Acta Neuropathol..

[102] Silveira-Mattos P.S., Narendran G., Akrami K., Fukutani K.F., Anbalagan S., Nayak K., Subramanyam S., Subramani R., Vinhaes C.L., Souza D.O. (2019). Differential Expression of CXCR3 and CCR6 on CD4+ T-Lymphocytes with Distinct Memory Phenotypes Characterizes Tuberculosis-Associated Immune Reconstitution Inflammatory Syndrome. Sci. Rep..

[103] Tibúrcio R., Narendran G., Barreto-Duarte B., Queiroz A.T.L., Araújo-Pereira M., Anbalagan S., Nayak K., Ravichandran N., Subramani R., Antonelli L.R.V. (2022). Frequency of CXCR3+ CD8+ T-Lymphocyte Subsets in Peripheral Blood Is Associated With the Risk of Paradoxical Tuberculosis-Associated Immune Reconstitution Inflammatory Syndrome Development in Advanced HIV Disease. Front. Immunol..

[104] Bell L.C.K., Noursadeghi M. (2018). Pathogenesis of HIV-1 and Mycobacterium Tuberculosis Co-Infection. Nat. Rev. Microbiol..

[105] Wilkinson K.A., Meintjes G., Seldon R., Goliath R., Wilkinson R.J. (2012). Immunological Characterisation of an Unmasking TB-IRIS Case. S. Afr. Med. J..

[106] Lawn S.D., Wainwright H., Orrell C. (2009). Fatal Unmasking Tuberculosis Immune Reconstitution Disease with Bronchiolitis Obliterans Organizing Pneumonia: The Role of Macrophages. AIDS.

[107] Bell L.C.K., Pollara G., Pascoe M., Tomlinson G.S., Lehloenya R.J., Roe J., Meldau R., Miller R.F., Ramsay A., Chain B.M. (2016). In Vivo Molecular Dissection of the Effects of HIV-1 in Active Tuberculosis. PLoS Pathog..

[108] Meintjes G., Stek C., Blumenthal L., Thienemann F., Schutz C., Buyze J., Ravinetto R., van Loen H., Nair A., Jackson A. (2018). Prednisone for the Prevention of Paradoxical Tuberculosis-Associated IRIS. N. Engl. J. Med..

[109] Drechsler H., Ayers C., Cutrell J., Maalouf N., Tebas P., Bedimo R. (2017). Current Use of Statins Reduces Risk of HIV Rebound on Suppressive HAART. PLoS ONE.

[110] Eckard A.R., McComsey G.A. (2015). The Role of Statins in the Setting of HIV Infection. Curr. HIV/AIDS Rep..

[111] Uthman O.A., Okwundu C., Gbenga K., Volmink J., Dowdy D., Zumla A., Nachega J.B. (2015). Optimal Timing of Antiretroviral Therapy Initiation for HIV-Infected Adults With Newly Diagnosed Pulmonary Tuberculosis: A Systematic Review and Meta-Analysis. Ann. Intern. Med..

[112] Shahani L., Hamill R.J. (2016). Therapeutics Targeting Inflammation in the Immune Reconstitution Inflammatory Syndrome. Transl. Res..

[113] Abay S.M., Deribe K., Reda A.A., Biadgilign S., Datiko D., Assefa T., Todd M., Deribew A. (2015). The Effect of Early Initiation of Antiretroviral Therapy in TB/HIV-Coinfected Patients: A Systematic Review and Meta-Analysis. J. Int. Assoc. Provid. AIDS Care.

[114] Meintjes G., Wilkinson R.J., Morroni C., Pepper D.J., Rebe K., Rangaka M.X., Oni T., Maartens G. (2010). Randomized Placebo-Controlled Trial of Prednisone for Paradoxical Tuberculosis-Associated Immune Reconstitution Inflammatory Syndrome. AIDS.

[115] Guidelines for Managing Advanced HIV Disease and Rapid Initiation of Antiretroviral Therapy. https://www.who.int/publications-detail-redirect/9789241550062.

[116] Durovni B., Cavalcante S. (2018). Preventive Therapy for HIV-Associated Tuberculosis. Curr. Opin. HIV AIDS.

[117] Hakim J., Musiime V., Szubert A.J., Mallewa J., Siika A., Agutu C., Walker S., Pett S.L., Bwakura-Dangarembizi M., Lugemwa A. (2017). Enhanced Prophylaxis plus Antiretroviral Therapy for Advanced HIV Infection in Africa. N. Engl. J. Med..

[118] Valin N., Pacanowski J., Denoeud L., Lacombe K., Lalande V., Fonquernie L., Girard P.-M., Meynard J.-L. (2010). Risk Factors for “unmasking Immune Reconstitution Inflammatory Syndrome” Presentation of Tuberculosis Following Combination Antiretroviral Therapy Initiation in HIV-Infected Patients. AIDS.

[119] Hopewell P.C., Reichman L.B., Castro K.G. (2021). Parallels and Mutual Lessons in Tuberculosis and COVID-19 Transmission, Prevention, and Control. Emerg. Infect. Dis..

[120] Pai M., Kasaeva T., Swaminathan S. (2022). COVID-19’s Devastating Effect on Tuberculosis Care—A Path to Recovery. N. Engl. J. Med..

[121] Song W.-M., Zhao J.-Y., Zhang Q.-Y., Liu S.-Q., Zhu X.-H., An Q.-Q., Xu T.-T., Li S.-J., Liu J.-Y., Tao N.-N. (2021). COVID-19 and Tuberculosis Coinfection: An Overview of Case Reports/Case Series and Meta-Analysis. Front. Med. (Lausanne).

[122] Zhou F., Yu T., Du R., Fan G., Liu Y., Liu Z., Xiang J., Wang Y., Song B., Gu X. (2020). Clinical Course and Risk Factors for Mortality of Adult Inpatients with COVID-19 in Wuhan, China: A Retrospective Cohort Study. Lancet.

[123] Wu C., Chen X., Cai Y., Xia J., Zhou X., Xu S., Huang H., Zhang L., Zhou X., Du C. (2020). Risk Factors Associated With Acute Respiratory Distress Syndrome and Death in Patients With Coronavirus Disease 2019 Pneumonia in Wuhan, China. JAMA Intern. Med..

[124] Chi Y., Ge Y., Wu B., Zhang W., Wu T., Wen T., Liu J., Guo X., Huang C., Jiao Y. (2020). Serum Cytokine and Chemokine Profile in Relation to the Severity of Coronavirus Disease 2019 in China. J. Infect. Dis..

[125] Seddiki N., French M. (2021). COVID-19 and HIV-Associated Immune Reconstitution Inflammatory Syndrome: Emergence of Pathogen-Specific Immune Responses Adding Fuel to the Fire. Front. Immunol..

[126] Cancio M., Ciccocioppo R., Rocco P.R.M., Levine B.L., Bronte V., Bollard C.M., Weiss D., Boelens J.J., Hanley P.J. (2020). Emerging Trends in COVID-19 Treatment: Learning from Inflammatory Conditions Associated with Cellular Therapies. Cytotherapy.

[127] Horby P., Lim W.S., Emberson J.R., Mafham M., Bell J.L., Linsell L., Staplin N., Brightling C., Ustianowski A., RECOVERY Collaborative Group (2021). Dexamethasone in Hospitalized Patients with COVID-19. N. Engl. J. Med..

[128] Salama C., Han J., Yau L., Reiss W.G., Kramer B., Neidhart J.D., Criner G.J., Kaplan-Lewis E., Baden R., Pandit L. (2021). Tocilizumab in Patients Hospitalized with COVID-19 Pneumonia. N. Engl. J. Med..

[129] Sandhu T., Tieng A., Chilimuri S., Franchin G. (2020). A Case Control Study to Evaluate the Impact of Colchicine on Patients Admitted to the Hospital with Moderate to Severe COVID-19 Infection. Can. J. Infect. Dis. Med. Microbiol..

[130] Merchant E.A., Flint K., Barouch D.H., Blair B.M. (2021). Co-Infection with Coronavirus Disease 2019, Previously Undiagnosed Human Immunodeficiency Virus, Pneumocystis Jirovecii Pneumonia and Cytomegalovirus Pneumonitis, with Possible Immune Reconstitution Inflammatory Syndrome. IDCases.

[131] Mertens J., Laghrib Y., Kenyon C. (2020). A Case of Steroid-Responsive, COVID-19 Immune Reconstitution Inflammatory Syndrome Following the Use of Granulocyte Colony-Stimulating Factor. Open. Forum. Infect. Dis..

[132] Garcia-Carretero R., Vazquez-Gomez O., Ordoñez-Garcia M. (2021). Delayed Immune Reconstitution Inflammatory Syndrome in an Immunosuppressed Patient With SARS-CoV-2. Cureus.

[133] Lalonde C.S., Zhuang T.Z., Aldredge A.A., Adelman M.W., AbouYabis A.N., McLemore M.L., Auld S.C. (2022). Abstract 444: COVID-IRIS: Immune Reconstitution after G-CSF Administration for Neutropenia during Acute COVID-19 Infection. Cancer Res..

